# Bioactivity of 2′-deoxyinosine-incorporated aptamer AS1411

**DOI:** 10.1038/srep25799

**Published:** 2016-05-19

**Authors:** Xinmeng Fan, Lidan Sun, Yun Wu, Lihe Zhang, Zhenjun Yang

**Affiliations:** 1 State Key Laboratory of Natural and Biomimetic Drugs, School of Pharmaceutical Sciences, Peking University, Beijing 100191, PR China; 2 Hubei Key Laboratory of Tumor Microenvironment and Immunotherapy, China Three Gorges University Medical College, Yichang, 443002 China

## Abstract

Aptamers can be chemically modified to enhance nuclease resistance and increase target affinity. In this study, we performed chemical modification of 2′-deoxyinosine in AS1411, an anti-proliferative G-rich oligodeoxynucleotide aptamer, which binds selectively to the nucleolin protein. Its function was augmented when 2′-deoxyinosine was incorporated at positions 12, 13, 15, and 24 of AS1411, respectively. In addition, double incorporation of 2′-deoxyinosine at positions 12 and 24 (FAN-1224dI), 13 and 24 (FAN-1324dI), and 15 and 24 (FAN-1524dI) promoted G-quartet formation, as well as inhibition of DNA replication and tumor cell growth, and induced S-phase cell cycle arrest. In further animal experiments, FAN-1224dI, FAN-1324dI and FAN-1524dI resulted in enhanced treatment effects than AS1411 alone. These results suggested that the position and number of modification substituents in AS1411 are critical parameters to improve the diagnostic and therapeutic function of the aptamer. Structural investigations of the FAN-1524dI/nucleolin complex structure, using molecular dynamics simulation, revealed the critical interactions involving nucleolin and 2′-dI incorporated AS1411 compared with AS1411 alone. These findings augment understanding of the role of 2′-deoxyinosine moieties in interactive binding processes.

Aptamers are short, synthetic single-stranded oligonucleotides that specifically bind to various molecular targets such as small molecules, proteins, nucleic acids, cells and tissues^1^. Known as chemical antibodies, these molecules can fold into complex three-dimensional structures and bind to target molecules. They are advantageous in being highly specific and relatively small in size and have low immunogenicity, thus offering the possibility of overcoming limitations of antibodies. Aptamers are generally selected using a biopanning method known as SELEX (Systematic Evolution of Ligands by Exponential enrichment), which was first reported independently by two groups in 1990^1^,^2^. Many reported aptamers are guanine-rich oligonucleotides (GROs) that play key roles in a wide range of human physiological processes *in vivo*. Chemically modified GROs can be used as research tools to study various biological mechanisms, including those involving aptamer–protein interactions. Scuotto^3^ developed acyclic nucleosides for aptamer modification as novel monomers to explore thrombin inhibition and found that both TT and TGT loops are involved in thrombin inhibition, but with different roles. Our previous work^4^, which incorporated *L*-isothymidine in a thrombin-binding aptamer (TBA), also suggested that TBA could interact with two thrombin molecules through the TT and TGT loops, although the second binding did not exhibit additional biological effects. Chemically modified GROs may also change activity. Scuotto^5^ reported that site-specific replacements of a single-loop nucleoside with a dibenzyl linker in the TBA sequence can preserve its anti-proliferative over anticoagulant activity. Taken together, these findings mean that suitable chemical modifications play important roles in aptamer studies.

AS1411 (Fig. 1A) is a 26-base GRO which targets nucleolin, a nuclear matrix protein found on the surface of many kinds of cancer cells and whose levels are known to be associated with the rate of malignant proliferation^6^, being elevated in rapidly dividing cells. Biological effects of AS1411 include inhibition of cellular proliferation and induction of cell death in many cancer cell types, but with minimal effects on normal cells^7^,^8^. A Phase І clinical trial of AS1411 has demonstrated no serious adverse effects, and AS1411 has now entered Phase II clinical trials.

Since AS1411 appears to have broad therapeutic potential^9^, considerable research has focused on improving its stability and biological properties, mainly through chemical modification. Numerous studies have worked on enhancing nuclease resistance of GROs and targeting affinity, with only a few reports specifically addressing AS1411. Terminal phosphorothioate (PS)-modified quadruplex oligonucleotides were found to have longer half-lives in bovine serum; in contrast, a single G→A mutation that was expected to disrupt quadruplex formation; with no full-length oligonucleotides, resulted in a dramatically decreased half-life in serum^9^. Moreover, at least three compounds with Cy3-labeled 5-(N-benzylcarboxyamide)-2′-deoxyuridine (5-BzdU) modification exhibited significantly increased targeting affinity to C6 cells^10^.

Results from our previous work^4^, which sought to identify structural modifications which can enhance AS1411 bioactivity through changing local space conformation, and not through G-quartet formation, showed most aptamers were found by the classic SELEX method, which amplified bound aptamers *via* polymerase chain reaction (PCR) to form a new, more refined aptamer library. PCR is used to amplify a specific region of a DNA strand, Thus, each deoxynucleoside in the DNA aptamer was selected from deoxyadenosine, deoxythymidine, deoxycytidine, and deoxyguanosine. In addition, there exists another type of naturally occurring nucleoside, 2′-deoxyinosine (2′-dI) (Fig. 1B), which is structurally similar to deoxyguanosine, apart from lacking the 2-amino group. 2′-dI can form two hydrogen bonds with each of the four naturally occurring nucleotide bases^11^ and is considered the first “universal” base; that is it can base-pair with all the natural bases and can hydrogen-bond with amino acid residues. Thus, chemical modification of 2′-dI offers a major advantage in clinical applications, as it is a naturally occurring base and non-immunogenic. In addition, with the incorporation of 2′-dI, the local spatial conformation of AS1411, particularly around the incorporation site, is changed, which, in turn, alters the bioactivity of the aptamer. The mode of recognition of nucleolin by 2′-dI-modified AS1411 is yet to be elucidated.

Therefore, to enhance the bioactivity of AS1411, the binding sites of all non-G-quadruplex structures were randomly substituted with 2′-dI. Circular dichroism (CD) spectra were collected to determine the effect of 2′-dI substitutions on the structure of the AS1411 modifications. Furthermore, to elucidate the AS1411 anti-proliferative activity, cell growth and DNA synthesis assays, and cell cycle distribution were performed. In addition, animal experiments were conducted to determine antitumor effects of 2′-dI substitutions *in vivo*. To improve our understanding of the structural determinants of 2′-dI in binding to nucleolin, molecular dynamics (MD) simulation was performed to examine the interactions of FAN-1524dI with the nucleolin RNA binding domain. The study aimed to investigate the interaction features between nucleolin and 2′-dI-modified AS1411, based on the dynamic structural information gained from MD studies. Knowledge of this molecular interaction mechanism will be essential for the rational design of a novel and potent aptamer.

## Results

### The function of four units of 2′-dI (FAN-12dI, FAN-13dI, FAN-15dI, FAN-24dI)-modified AS1411 aptamers was improved

To screen for suitable modified sites, the sites which were not in the formation of G-quadruplex were randomly substituted with 2′-dI. Oligonucleotide sequences used in this and later experiments are shown in Table 1 and Supplementary Table S1. To detect the antitumor active of four units 2′-dI-modified AS1411 aptamers *in vitro*, DNA synthesis assays, cell growth assays, and flow cytometry analyses of propidium iodide-stained cell nuclei were performed.

Results of the EdU incorporation assay showed that MCF-7 human breast cancer cells treated with FAN-12dI, FAN-13dI, FAN-15dI and FAN-24dI (Supplementary Fig. S1) had enhanced ability to inhibit DNA replication. In addition, we tested the effects of 2′-dI-modified AS1411 on the growth of MCF-7 cells in culture. FAN-12dI, FAN-13dI, FAN-15dI and FAN-24dI (Supplementary Fig. S2) showed a greater decrease in proliferative rate, compared with AS1411 control, while the activity of FAN-9dI, FAN-18dI and FAN-21dI was down-regulated considerably. Furthermore, there were larger number of cells in S-phase after treatment with FAN-12dI, FAN-13dI, FAN-15dI or FAN-24dI, with changes to varying degrees in the cell cycle distribution, compared with AS1411 (Table 1, Supplementary Fig. S3).

All three experiments conducted suggested that aptamer function was improved when 2′-dI was incorporated at appropriate positions in AS1411. Better biological effects were also obtained, compared with AS1411.

### Double incorporation of 2′-dI in AS1411 had stable chemical property

**Figure 1:**
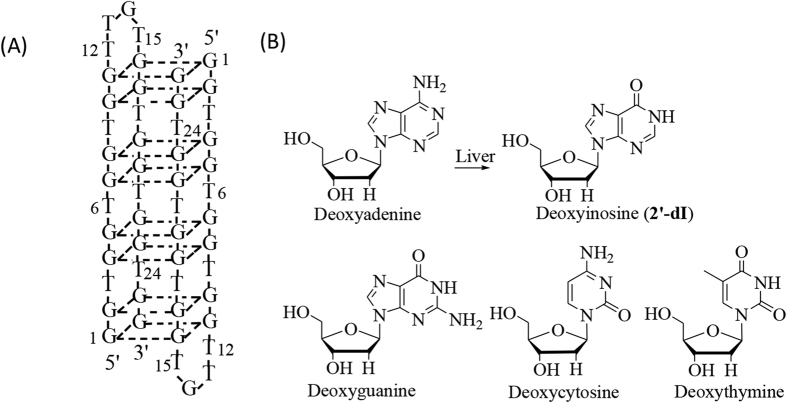
(**A**) Secondary structure sketch map of AS1411. (**B**) Chemical structures of 2′-deoxynucleosides.

From analyses of the sketch map of the secondary structure of AS1411 (Fig. 1), we observed that positions 12, 13 and 15 were located in the loop, whereas position 24 was found distant from this region. Therefore, incorporating 2′-dI at positions 12, 13, 15 and 24 simultaneously may either induce too much conformational changes or lead to crowding, that would decrease binding affinity to nucleolin. Indeed, our experimental results lend support to this. The same cell proliferation experiment detected bioactivity of AlldI (Supplementary Table S1, four sites of AS1411 were incorporated with 2′-dI at the same time), result showed that (Supplementary Fig. S4) compared with AS1411, there were increased viable cell numbers. These finding showed that the bioactivity of AlldI did not improve and was even lower than natural AS1411.

Furthermore, a previous study^9^ showed incorporation of 5-BzdU at position 24 significantly increased the affinity of aptamer to cancer cells. To further optimize AS1411, our next modification strategy involved double incorporation of 2′-dI, that is one 2′-dI incorporation fixed at position 24 and the other incorporation at positions 12, 13 or 15 (FAN-1224dI, FAN-1324dI and FAN-1524dI; Table 1). The aim was to further improve the biological effects on the basis of 2′-dI-modified AS1411, respectively.

CD spectra provide reliable information for identifying DNA structures and thus are useful for characterizing the G-quadruplex structure. It is generally thought that G-quartets^12^, essential for the maintenance of AS1411 activity, on CD analysis, show positive ellipticity maximum at 264 nm and negative ellipticity minimum at 240 nm^13^. It is worth noting that the structural features of AS1411 had not been investigated sufficiently, some studies showed antiparallel-type G-quadruplexes, including AS1411, exhibiting maximum at 295 nm and a minimum at 265 nm^14^,^15^. Moreover, other studies reported that AS1411 produced at least eight monomeric quadruplexes with a high degree of polymorphism^16^. We believed that, after full annealing, AS1411 would be in a state of partial equilibrium.

**Figure 2:**
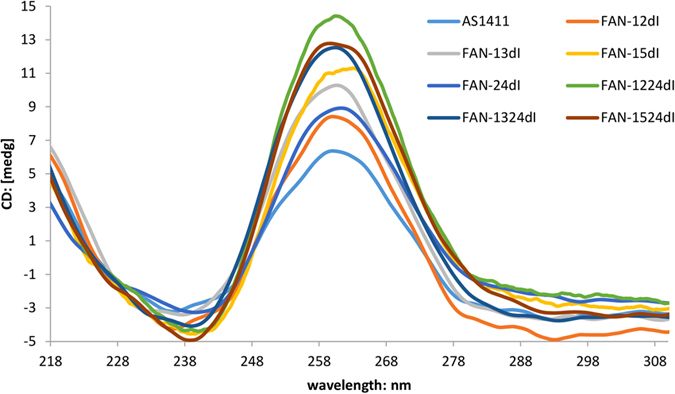
CD spectra of 2′-dI modified AS1411. CD data is obtained with a 5 μM concentration in the presence of in 10mM sodium phosphate buffer, pH 7.0, containing 0.1 M KCl. All aptamers are boiled for 5 min, and anneal at 60 °C for 50 h.

Data from CD spectra for AS1411 and 2′-dI-modified AS1411 were obtained to investigate the impact of 2′-dI on the overall structure of AS1411. The findings correlated with the predicted results (Fig. 2), that is, all three modified aptamers FAN-1224dI, FAN-1324dI and FAN-1524dI had a higher positive ellipticity maximum in the CD spectra, and the intensity of the CD bands was increased, particularly the positive band at around 264 nm. This difference was most likely due to 2′-dI contributing to stabilize G-quartets when interacted with adjacent bases, whereby the adhesion changed the local space conformation to stable G-quartet formation. In the absence of nucleolin, the modified AS1411 adopt a stable conformation and the stability of G-quadruplex structure is dominated by the interactions between the adjacent bases. These observations reflect the stabilization of the G-quadruplex by 2′-dI when incorporated at the correct positions and in the appropriate quantities.

AS1411 with antiparallel-type G-quartets was resistant to degradation when placed in serum-containing medium . Serum degradation assay had performed to ensure aptamer stability. Results show that (Fig. S5), all AS1411 and 2′-dI modified AS1411 remained after 3 days in 10% serum-containing medium, the experimental conditions was coincident with that in cellular level. The results indicated that the improvement of 2′-dI modified AS1411 was due to structural change, not the improvement of anti-enzymatic ability.

### Double incorporation of 2′-dI in AS1411 further enhanced aptamer biological effects

Previous studies have reported that AS1411 can inhibit DNA replication^7^. Thus, to investigate the effects of 2′-dI-modified AS1411 on cellular proliferation, we analyzed DNA replication in cells treated with aptamers. This was achieved by determining the incorporation of EdU, a nucleoside analog of thymidine that is incorporated into DNA during active DNA synthesis by proliferating cells only. After EdU incorporation, a fluorescent molecule was added that reacted specifically with EdU, such that fluorescent proliferating cells could be visualized^17^.

**Figure 3:**
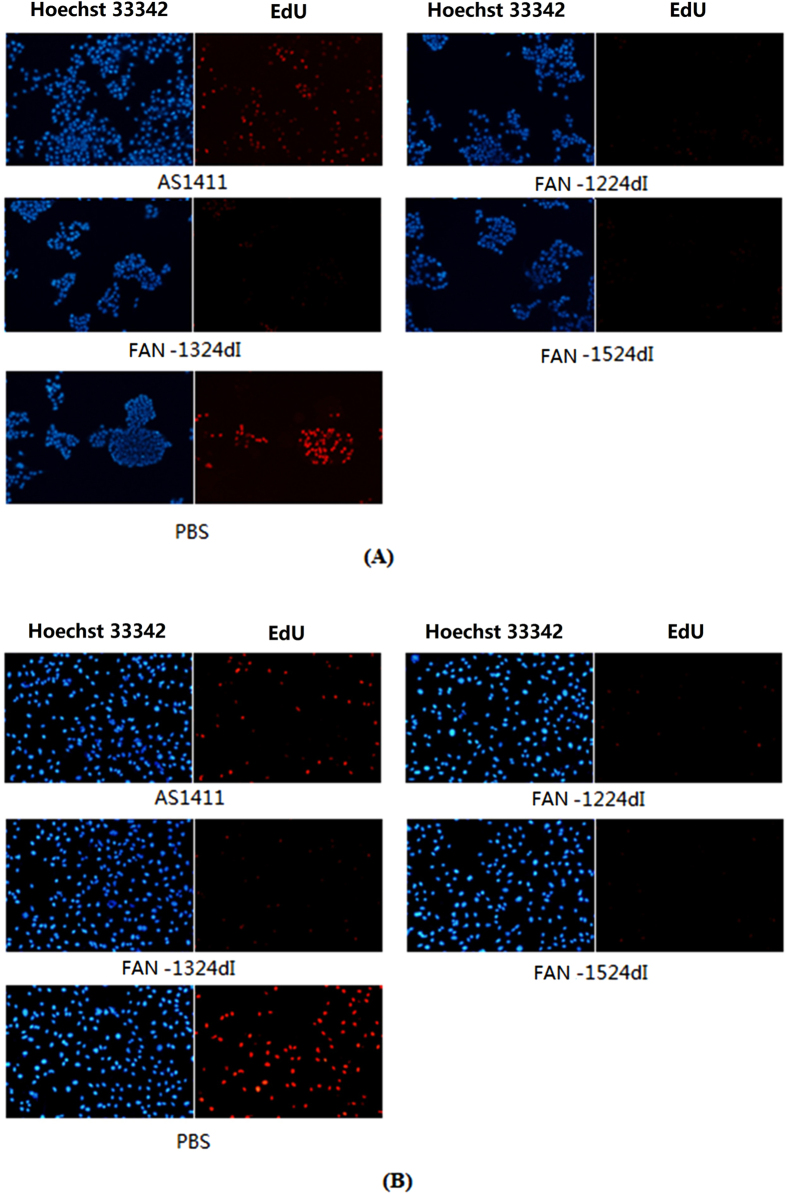
DNA synthesis in untreated 2 kind of cells (PBS as control) and cells treated with AS1411 (control oligonucleotide) or FAN-1224dI/FAN-1324dI/FAN-1524dI (active oligonucleotide). Cells were treated a final concentration of 18 μM for 72 h and then expose to 50 μM EdU for 2 h at 37 °C. DNA was stained with 5 μg/mL Hoechst 33342 (50 μl per well) for 30 min and imaged under a fluorescent microscope. (**A**) MCF-7 cells. (**B**) MDA-MB-231 cells.

DNA synthesis assays (Fig. 3) showed that, in both MCF-7 and MDA-MB-231 cells, FAN-1224dI, FAN-1324dI and FAN-1524dI led to a considerable reduction in red staining; weaker staining by EdU indicated that less *de novo* DNA synthesis occurred in these cells. The data presented suggested that FAN-1224dI, FAN-1324dI and FAN-1524dI all had enhanced ability to block DNA replication in human breast cancer cell lines.

**Figure 4:**
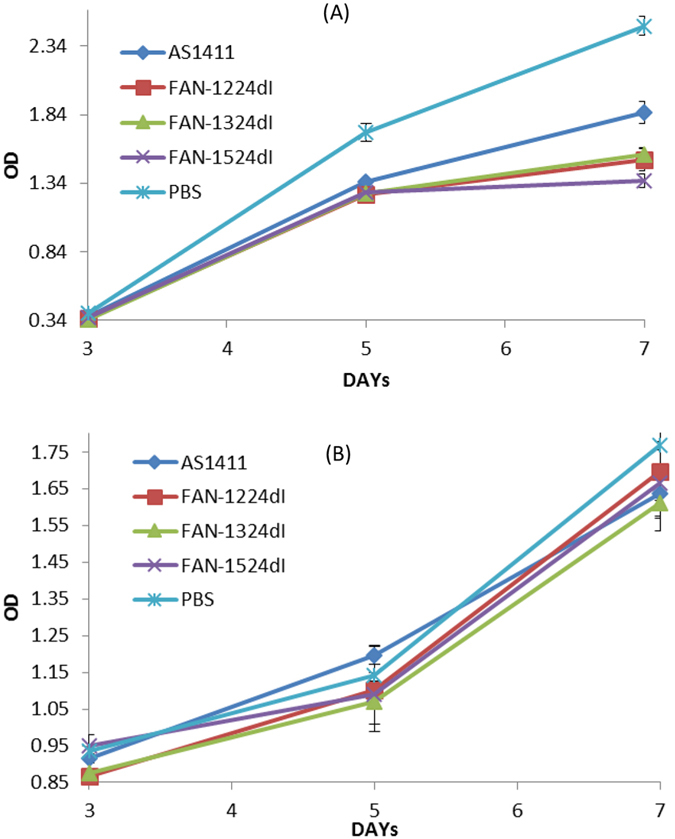
CCK-8 assays showing the growth of MCF-7 cells (**A**) and 293 cells (**B**) treated with FAN-1224dI, FAN-1324dI, FAN-1524dI or PBS as a control. oligonucleotides (or PBS as control) are added directly to the culture medium to give a final concentration of 7.5 μM (day 1). On days 2-4 further oligonucleotide equivalent to half the initial dose is added. Cells are assayed using the cell counting kit-8 (CCK-8) (Dojindo Laboratorie, Japan) on 3, 5, 7 days after treatment. The OD_450 _nm value is proportional tothe number of viable cells in the sample.

The ability of AS1411 to inhibit DNA replication is correlated with the ability to inhibit cellular proliferation^12^. We tested the effects of 2′-dI-modified AS1411 on the growth of MCF-7 cells in culture. In our study, we detected the antitumor effect from day 3, which is consistent with a previous study reporting that AS1411 induction of cell death occurred only after about 72 h of exposure to AS1411^9^. Figure 4 shows the results of CCK-8 assays which determined the relative viable cell numbers in the treated cell line. MCF-7 cells treated with FAN-1224dI, FAN-1324dI or FAN-1524dI had fewer viable cell numbers, compared with treatment with AS1411 control, but with minimal effects on normal cells. Examination of the growth-inhibited cells suggested that FAN-1224dI, FAN-1324dI and FAN-1524dI can each suppress human breast cancer cellular proliferation more potently than control treatments.

Previous studies showed that the ability of oligonucleotides to inhibit cellular proliferation is correlated with their ability to induce S-phase cell cycle arrest^12^. The gradual arrest of cells in S-phase may be related to the rate of oligonucleotide uptake, which is thought to be relatively slow. To investigate cell cycle perturbations induced by AS1411 and its 2′-dI analogs, flow cytometry analysis of propidium iodide-stained nuclei was performed. We observed an increase in the fraction of MCF-7 cells in S-phase of the cell cycle for cells treated with FAN-1224dI, FAN-1324dI or FAN-1524dI, which was considerably higher than cells treated with FAN-12dI, FAN-13dI, FAN-1524dI, FAN-24dI or AS1411; similar results were obtained with MDA-MB-231 cells (Table 1, Supplementary Fig. S3). These findings, which were consistent with our cell growth assay results, indicated that FAN-1224dI, FAN-1324dI and FAN-1524dI exhibit a stronger ability to influence the different phases in tumor cell cycle and induce S-phase cell cycle arrest, and further confirm the advantages of our modification strategy in terms of improving aptamer biological properties. Taken together, these results showed that this modification strategy was more effective in maintaining the chemical stability and biological effects of AS1411.

### FAN-1224dI, FAN-1324dI and FAN-1524dI increased the specific affinity to nucleolin

AS1411 targets nucleolin, an abundant multifunctional 110 kDa phosphoprotein^15^ and one of the major nucleolar proteins that functions as a shuttle protein between the nucleus and cytoplasm; it is also found on the many kinds of cancer cells surface where it acts as a binding protein for a variety of ligands implicated in cellular proliferation, differentiation, adhesion, mitogenesis and angiogenesis.

SPR was used to study the binding force with nucleolin. Table 1 shows the K_D_ values of natural AS1411 and doubly-incorporated 2′-dI-AS1411. We only tested the doubly-incorporated 2′-dI-AS1411, which exerted higher bioactivity in the CCK-8 assay. FAN-1224dI, FAN-1324dI and FAN-1524dI each revealed considerable enhancements in target affinity, thus indicating that our modification strategy improved binding interactions of AS1411 at the molecular level.

**Figure 5:**
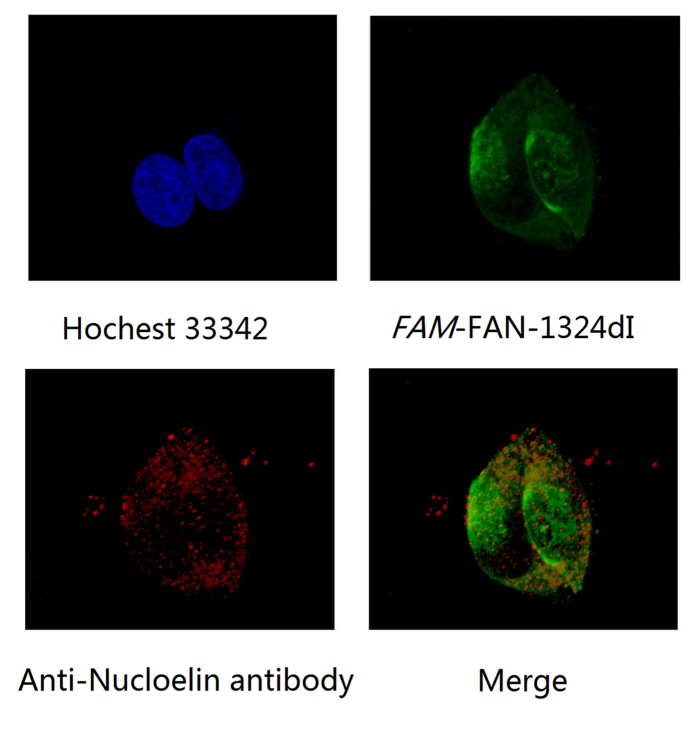
Immunofluorescence images of MCF-7 cells incubate with anti-nucleolin antibody at 4 °C overnight and 550 nM FAM-FAN-1324dI for 30 min at 37 °C. Permeabilize cells with 0.5% (v/v) Triton X-100, cells are then stained with 5 μg/mL Hoechst 33342 (50 μL per well) for 30 min, Confocal images are obtained using NIS-Elements and Bitplane imaris software. Antibody in red and FAM-FAN-1324dI in green, nucleolus are shown in blue.

Applying the principle of specific binding between an antibody and its antigen, we determined the accurate localization of modified AS1411 in MCF-7 cells. Immunofluorescence localization (Fig. 5) in *FAM*-FAN-1324dI using antinucleolin antibody indicated that FAN-1324dI was bound specifically to nucleolin. This evidence indicated that the increase in aptamer bioactivity was not attributed to nonspecific binding, and the modification strategy improved aptamer function without affecting binding specificity.

### FAN-1224dI, FAN-1324dI and FAN-1524dI resulted in stronger growth suppression effects in MCF-7 xenografts, compared with AS1411

**Figure 6:**
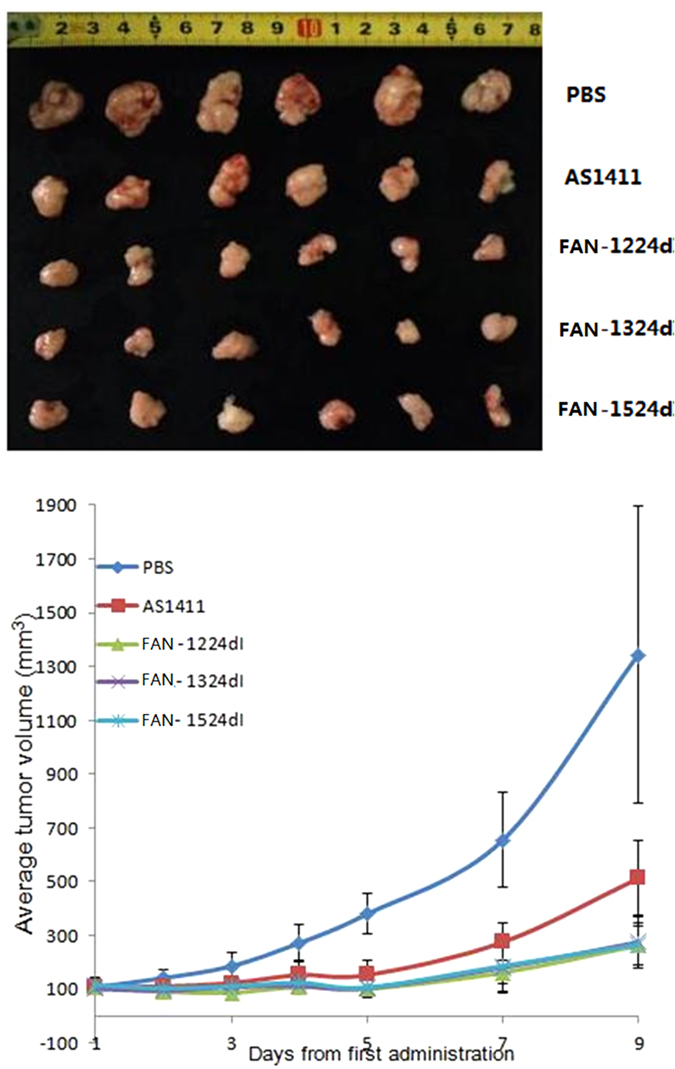
FAN-1224dI, FAN-1324dI and FAN-1524dI suppresses growth of MCF-7 xenografts.

Since our *in vitro* studies showed a suppression in human breast cancer cellular proliferation and viability, we next evaluated these effects *in vivo*. To this end, FAN-1224dI, FAN-1324dI and FAN-1524dI activities were confirmed in nude mice harbouring MCF-7 xenografts. As shown in Fig. 6, the effect of aptamers on cancer growth was confirmed by a delayed increase in the volume of the cancers. Average tumor volumes in the FAN-1224dI, FAN-1324dI and FAN-1524dI groups were consistently and significantly lower than in the control group (P value < 0.01 vs AS1411 control). We showed that FAN-1224dI, FAN-1324dI and FAN-1524dI suppressed human breast cancer growth, both *in vitro* and *in vivo*, with significantly altered levels of AS1411. The results shown in Fig. 6 are the mean of six mice for each group.

### Structure variations are present in the FAN-1524dI/nucleolin complex

**Figure 7:**
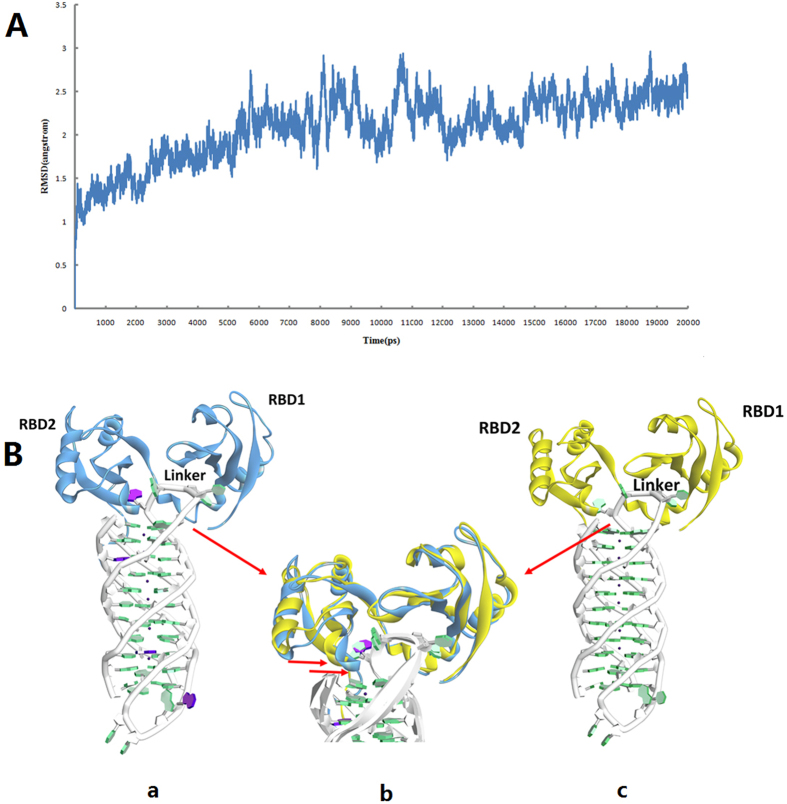
(**A**) RMSD curves for the whole complex structure during the MD simulation. (**B**) The overall structure and structure comparation of FAN-1524dI-RBD12 and AS1411-RBD12. (a) The average structure of FAN-1524dI-RBD12 from MD. The protein and FAN-1524dI are colored blue and green, respectively. 2′-dI at position 15 is colored purple; (b) Superimposition of FAN-1524dI-RBD12 (blue) and AS1411-RBD12 (yellow) structures. The red arrow depicts the protein rotation; (c) The complex structure used for constructing the initial FAN-1524dI-RBD12 for MD and the protein and AS1411 are colored yellow and green, respectively.

The interaction of FAN-1524dI with RBD12 and the influence of 2′-dI on the binding mode were examined by analyzing MD trajectories. To assess the overall structural stability of the FAN-1524dI–RBD12 complex, root mean-square deviation (RMSD) profiles, with the initial structure as reference, were analyzed along the trajectories. As shown in Fig. 7A, the RMSD for the whole structure slowly increased during the initial 6 ns of simulations and then tended to converge, indicating the systems were stable and equilibrated. Of note, the RMSD displayed oscillations from 8 to 12 ns. Visual inspection of the trajectories clearly showed that the mobility was attributed to the flexibility of two loop regions of RBD12 which should be connected to the other domain of nucleolin. Actually, we also have observed the similar conformation changes in FAN-15dI-RBD complex simulation. However, these fluctuations did not have any significant impact on the structure of the binding site. After the trajectory had reached equilibrium, the RMSD increased to 2.5 Å, which indicated that FAN-1524dI formed a stable complex structure with RBD12.

### Overall structure and binding mode analysis

The complex structure formed between FAN-1524dI and RBD12 represents a paradigm in the understanding of how FAN-1524dI binds to nucleolin. The whole averaged structure over the last 5 ns of molecular dynamics simulations was extracted (Fig. 7A). After the simulation, the FAN-1524dI formed a antiparallel G-quadruplex similar to natural AS1411. Comparison between RBD12 complexed with AS1411 (Fig. 7C) and the FAN-1524dI–RBD12 complex revealed several differences. Thus, a noticeable rotation of the whole protein structure was observed in the FAN-1524dI complex. As shown in Fig. 7B, the protein underwent significant movement towards the central core of G-quartet giving rise to a more compact and stable complex. However, the manner in which RBD12 recognized AS1411 and FAN-1524dI was very similar, with only the backbones of the G-quadruplex in slightly different conformations in the two complexes. In the FAN-1524dI–RBD12 complex (Fig. 7A), both the RBD domains and the 12-residue linker interacted extensively with the FAN-1524dI loop region (TTGdI). It was worthy noting that only the 2′-dI at position 15 was involved in the binding interface, whereas, the 2′-dI at position 24 formed a more stable stacking interactions with the adjacent G-quartets. Our aim was to design a type of chemical modification that enhances aptamer bioactivity through changing local conformation, and not G-quartet formation. These findings are in agreement with our previously described CD results showing that FAN-1524dI can fold into a native-like antiparallel G-quadruplex structure. According to the computational model, this kind of modification at position 24 did not have direct contributions to the binding mode, but the stronger stacking interactions can increase the stability of the modified G-quadruplex. This model also provided a good explanation for the stability results of FAN-1524dI and FAN-15dI by the CD spectrum.

**Figure 8:**
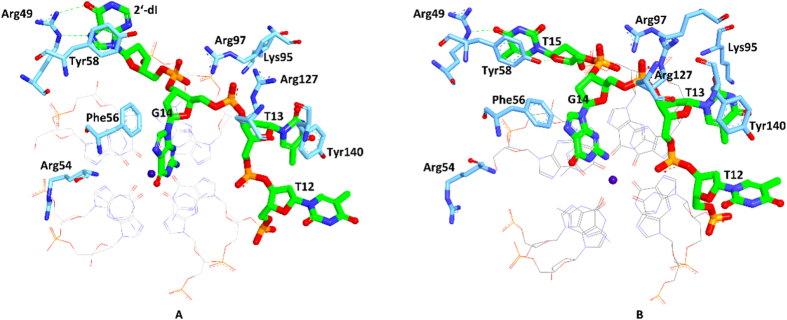
Binding mode analysis of FAN-1524dI-RBD12 (**A**) and AS1411-RBD12 (**B**). In both panels, residues (blue) and nucleotide (green) making favorable interactions are represented as solid sticks with black labels. Green dots represent hydrogen bonds.

To further investigate FAN-1524dI interactions in the binding process, we analyzed the binding mode taken from the last 4 ns MD trajectory. Visual inspection of the complex revealed critical interactions involving RBD12 and FAN-1524dI (Fig. 8A,B): (a) the π − π stacking interactions were formed between G14 and Phe56, and between T13 and Tyr140; (b) the NH1 and NH2 atoms of Arg97 formed ion pairs with O1P and O2P atoms of 2′-dI, respectively; (c) the side chain of Arg127 formed an ion pair with O2P atom of G14; (d) the NZ atom of Lys95 formed an ion pair with O1P atoms of G17; (e) a hydrogen bond was observed between the side of Arg49 and the base at position 15. These interactions were conserved both in the natural and modified systems. These results indicated the binding mechanism of nucleolin and FAN-1524dI is dominated by hydrophobic and electrostatic interactions. Moreover, the loop region played an important role in the binding process. In addition, these results also confirmed that the initial computational model is reasonable and can be used for other studies.

Despite the binding mode not causing drastic structural changes upon 2′-dI substitution, these changes led to new interactions with RBD12. In particular, Arg54 was initially not in contact with AS1411. However, in FAN-1524dI, a strong ion pair interaction was established with G26. Additionally, 2′-dI formed stronger hydrophobic interactions with Tyr58. Furthermore, a new hydrogen bond was observed between the side chain of Arg49 and 2′-dI. As expected, the most evident conformational variation was 2′-dI. These additional interactions provided a good explanation for the improved binding affinity of FAN-1524dI.

## Discussion

Aptamer science is now flourishing, and aptamers continue to develop as reliable clinical agents. Aptamers represent a unique class of molecules that are larger than small-molecule drugs, but smaller than antibodies. They have unique pharmacokinetic properties and are non-immunogenic^18^. Although research has made some progress in the field of aptamers, there are still no new aptamers approved for sale since Macugen^®^, the first aptamer compound approved for clinical use in December 2004. Most candidate drugs only reach preclinical or early clinical investigation. This is because non-modified aptamers have a half-life ranging from minutes to hours, and, with an inherently low molecular weight, they are cleared rapidly from the bloodstream, mainly due to nuclease degradation and renal clearance from the body. Therefore, the search for a generic modification strategy to solve the above problems has become a hot topic for future research. Based on the data presented in this study, incorporation of 2′-dI as a new aptamer modification can maintain chemical stability and promote biological effects through changing the local space conformation, rather than by G-quartet formation.

In this study, a modification method was applied employing 2′-dI which harbors alternative nucleobases and occupies a larger steric structure. Our findings showed that 2′-dI significantly increased desirable aptamer activities. We did not change the guanine bases in the backbone of the G-quadruplex structure, as these are highly conserved sequences^9^. The loop region plays a key role in the process of protein binding. If modified appropriately, the activity of many sequences is increased, such as with FAN-12dI, FAN-13dI and FAN-15dI; however, when the loop region was changed too much, the activity decreased. Besides the loop region, other positions also play a critical role in target protein binding, including position 24. Thus, this illustrates AS1411 has more binding sites targeting nucleolin.

The conformation of AS1411 should be different when AS1411 existed on its own and in the binding process to nucleolin. We described two different conformation states of 2′-dI incorporated AS1411: the stable conformation(CD analysis) and the binding conformation (AS1411-nucleolin complex model). CD analysis showed that the intensity of the CD bands was increased, particularly the positive band at around 264 nm, we believed the main reason of the changes was 2′-dI interacted with adjacent G-quartet, improved the ability to form stable structure. Based on the constructed modified AS1411-nucleolin complex model, we tried to reveal the critical interactions involving nucleolin and modified AS1411. Obviously, the position of 2′-dI 15 indicated no stacking interaction with an adjacent base, however, these results do not conflict with the CD results. Albeit the formation of G-quadruplex is a prerequisite for the binding process, however, the flexibility of conformation of loop region is a common phenomenon during the binding process. In some cases, there is not a significantly positive correlation between the stability and bioactivity. In order to form more interactions with nucleolin, the loop region should adopt an more extended conformation, thus the interactions of adjacent bases existed in the stable conformation is disrupted. Binding mode analysis also shown that 2′-dI led to new interactions with nucleolin and formed stronger hydrophobic interactions and ion pair interaction. To our knowledge, this is the first study describing the unambiguous binding mode and interactions between FAN-15dI and nucleolin has been revealed using a computer simulation technique.

Aptamers play various key roles in many aspects of biology. Tan *et al*.^19^ proposed that stabilization of telomere G-quadruplex by a chemical ligand could accelerate telomere shortening in proliferating cells. Scuotto^5^ showed that replacing one residue in the TT or TGT loops in TBA with a dibenzyl linker may switch the activity of TBA from anticoagulant to anti-proliferative. Our findings here showed a clear improvement in the activity of our 2′-dI-modified AS1411. We will conduct further research to study the behaviour of 2′-dI-modified AS1411 in tumour-related gene networks, with a view to developing it into an important bioprobe.

Early diagnosis of cancer is crucial to patient prognosis and survival and may lead to cancer successful treatment. Therefore, high-specificity affinity aptamers need to be developed for early cancer diagnosis, which can be achieved through chemical modification. Binding affinity analyses showed FAN-1224dI, FAN-1324dI and FAN-1524dI have enhanced ability for identifying their target proteins. Thus, such chemically modified aptamers could be used as valuable clinical tools in the identification of cancer at very early stages of the disease and in the evaluation of cancer therapy.

Conventional cancer treatment includes surgery, radiotherapy and chemotherapy^20^. Aptamers, as a new class of therapeutics and drug-targeting entities, possess numerous advantages over other therapeutic modalities. With appropriate design and enhancing modifications, it is possible to construct the desired pharmacokinetic and biodistribution profile for a specific aptamer intended for a specific clinical application. In this study, double 2′-dI incorporation was used as our modification strategy to improve AS1411 properties. Using experiments at the cellular level and mouse studies, we confirmed that changing local space conformation enhanced aptamer bioactivity and improved specificity and affinity - all properties that make aptamers such promising therapeutic agents^5^.

## Methods

### Cell culture and treatment

Cells were cultured in a humidified incubator maintained at 37 °C in 95% air/5% carbon dioxide. MCF-7 human breast cancer cells were cultured in Dulbecco minimum essential medium (DMEM, M&C Gene Technology), supplemented with 10% fetal bovine serum (PAA). MDA-MB-231 human breast cancer cells and HEK-293 were cultured in DMEM (M&C Gene Technology), supplemented with 10% fetal bovine serum (HyClone, Thermo Scientific). All experiments were performed on cells in the exponential growth phase. AS1411, with sequence 5′-(GGT GGT GGT GGT TGT GGT GGT GGT GG)-3′, *FAM*-FAN-AS1411, and all AS1411 constructs randomly substituted with 2′-dI sequences were from Invitrogen Ltd. AS1411 was dissolved in phosphate buffered saline (PBS) (M&C Gene Technology), before being added to cell cultures.

### Circular dichroism study

Oligonucleotides, at a final concentration of 5 μM, were resuspended in 10 mM sodium phosphate buffer (pH 7.0, containing 0.1 M KCl), heated for 5 min, and annealed at 60 °C for 50 h^14^. Samples were analyzed using a J-610 spectropolarimeter (Jasco) using 0.5 ml quartz cuvettes with a 2 mm path length.

### Serum Degradation Assay

Serum degradation assays were performed by incubating 1 μL of 20 μM aptamers in DMEM supplemented with 10% fetal bovine serum at 37 °C. Aliquots of 10 μL were collected after being treated for 3 days, then the solution was immediately frozen in liquid nitrogen and stored at −80 °C until analysis. Together with 3 μL of 6× DNA loading buffer, all the samples were resolved in 20% polyacrylamide gels and visualized by SYBR.

### DNA synthesis assay

Cells were seeded in 96-well plates at a density of 1.5 × 10^3^ cells per well, incubated for 16 h and then treated by direct addition of oligonucleotides to the culture medium at a final concentration of 18 μM (untreated samples were supplemented with an equal volume of PBS). At 72 h post-treatment, cell counts were determined using the 5-ethynyl-2′-deoxyuridine (EdU) incorporation assay (Ribobio). Cells were exposed to 50 μM EdU for 2 h at 37 °C, then fixed in 4% formaldehyde for 30 min at room temperature, and then permeabilized in 0.5% Triton X-100 for 10 min. Cells were washed with PBS, and each well was incubated with 100 μL of 1 × Apollo^®^ reaction cocktail (Ribobio) for 30 min. DNA was then stained with 5 μg/ml Hoechst 33342 (50 μL per well, Ribobio) for 30 min and visualized under a fluorescence microscope (Olympus IX81).

### Cell growth assay

Cells were plated in 96-well plates at a density of 1.5 × 10^3^ cells per well in the appropriate serum-supplemented medium and incubated for 16 h for cell adherence. Oligonucleotides (or PBS as control) were then added directly to the culture medium to a final concentration of 15 μM (day 1). On days 2–4, further oligonucleotides, equivalent to half the initial dose, were added. Cells were assayed using the cell counting kit-8 (CCK-8) (Dojindo Laboratories) several days after plating. The culture medium was not changed throughout the duration of the experiment.

### Flow cytometry analysis of cell cycle

Cells were plated in 6-well plates at a density of 10^5^ cells per well. After incubation at 37 °C for 8 h for cell adherence, cells were treated by direct addition of oligonucleotides to the culture medium to a final concentration of 20 μM. At 72 h post-treatment, cells were harvested by trypsinization and washed in PBS, fixed in cold 70% ethanol for 2 h at 4 °C, then treated the cells with RNase A (M&C Gene Technology) and stained with propidium iodide (M&C Gene Technology). Cells were then analyzed using FACSCalibur™ cytometer (BD Biosciences). The percentage of cells in G_1_/G_0_, S and G_2_/M phases was determined using the Modfit program (Verity Software House).

### Surface Plasmon Resonance (SPR)

SPR experiments were performed employing a BIAcore 3000 model (GE Healthcare) at 25 °C. Nucleolin protein (Abcam) was immobilized on CM5 sensor chips using PBS-EP (0.01 M HEPES, 0.15 M NaCl, 3 mM EDTA, and 0.005% Surfactant P20, pH 7.4, filtered and degassed before use). Aptamer was injected to a pre-activated sensor chip surface for 45 s at a flow rate of 30 μL/min. After each run, the surface was regenerated with 5 mM NaOH for 10 s at 30 μL/min.

### Confocal microscopy

MCF-7 cells were grown on confocal observation dishes for 18 h. The culture medium was removed and cells fixed by 100 μL 4% formaldehyde and incubated on ice for 5 min. To stain cell surface nucleolin, cells were pre-incubated with blocking buffer (5% bovine serum albumin in PBS) for 1 h, washed twice with PBS, and then incubated with anti-nucleolin antibody (Abcam) at 4 °C overnight. Cells were then washed twice with PBS and incubated with Alexa Fluor 647-conjugated anti-rabbit immunoglobulin G (IgG, Life technologies) (1:1000) for 1 h at 37 °C. Cells were washed three times and then incubated with 550 nM *FAM*-FAN-1524dI for 30 min at 37 °C. The cells were again washed three times in PBS and then permeabilized with 0.5% (v/v) Triton X-100 in PBS buffer for 10 min. Cells were stained with 5 μg/ml Hoechst 33342 (50 μL per well) for 30 min and observed under an A1Rsi confocal microscope (Nikon Instruments Inc.). Confocal images were obtained using NIS-Elements and Bitplane imaris software (Nikon Instruments Inc.).

### Animals and tumor growth model

Bilaterally ovariectomized female athymic nude mice (Balb/c/Nu/Nu), aged 4 weeks, were purchased from the Department of Laboratory Animal Science (BJMU) and housed in a dedicated nude mouse facility with micro-isolator caging and handled in a unidirectional laminar airflow hood. Xenografts were established via subcutaneous inoculation of MCF-7 cells at 200 μL (5 × 10^6^ cells/200 μL) per site into the right side of the back of nude mice. Five days after inoculation, when tumor volume reached approximately 100 mm^3^, mice were randomly divided into five groups (*n* = 6): blank (PBS), control (AS1411), 1224dI (FAN-1224dI), 1324dI (FAN-1324dI) and 1524dI (FAN-1524dI). Mice with established tumors were treated by intraperitoneal injection of aptamers at doses equivalent to approximately 3 ODs per mouse per day, daily for the first 5 days, with additional treatments at day 7, for a total of six injections. The volume was calculated using the formula [1/2 × L × W^2^]^21^, where length (L) and width (W) were measured using a caliper before aptamer administration. All procedures involving experimental animals were performed in accordance with protocols approved by the Committee for Animal Research of Peking University, China, and conformed to the Guide for the Care and Use of Laboratory Animals (NIH publication No. 86-23, revised 1985).

### Initial models

To our knowledge, there are no reports of structures of AS1411 or the AS1411–nucleolin complex model system solved by X-ray crystallography or nuclear magnetic resonance (NMR). The initial dimeric model of the antiparallel AS1411 structure was constructed using the human telomere solution structure. The aptamer GRO29A used in this study had a 5′-terminal “tail” (5′-TTT). However, a previous study showed that 5′-TTT of GRO29A was not essential for activity^14^. For the nucleolin structure, a previous study showed that the two RNA-binding domains (RBD12) are responsible for interaction with AS1411^22^. Thus, in the absence of experimental data for the whole structure of nucleolin, the available X-ray structure RBD12 (PDB ID:2KRR) was selected in the present study. In order to obtain the complex structure of natural AS1411 and RBD12, rigid body docking calculations were carried out, using the program ZDOCK in Discovery studio 2.5 software package (Discovery Studio 2.5, Accelrys Inc.). Moreover, fully solved MD calculations of both AS1411 and the AS1411–nucleolin complex have been completed successfully and will be reported separately. The AS1411–nucleolin complex is described below.

For subsequent MD simulations, we selected one representative structure, FAN-1524dI-modified AS1411 because, based on computational models and available published literature, the binding site and critical interactions are mediated by the loop region of AS1411^10^. Importantly, FAN-1524dI exhibited the best binding affinity among all the modifications. Thus, this allowed us to investigate computationally the effects of 2′-dI on the conformation and dynamics of the complexes. Since structural data were also not available in the case of the modified aptamer, as a template, the preceding AS1411–RBD12 computational model was modified to produce the starting model of the FAN-1524dI–RBD12 structure.

The force field parameters for 2′-dI were obtained by quantum chemical methods using the Gaussian 09 program (Gaussian 09, Gaussian, Inc.). At the B3LYP/6-31G* level of theory, we optimized the entire geometry and calculated the HF/6-31G* electrostatic potential. The RESP strategy was used to obtain the partial atomic charges. The non-standard residue in FAN-1524dI was created using the Xleap module (AMBER 11). The model was explicitly solved in a rectangular box which extended 10 Å away from any solute atom; potassium ions were added to provide electroneutrality.

### Molecular dynamics simulation

MD simulation was performed using the AMBER 11 software package utilizing the all-atom force field AMBER99SB (AMBER 11). Initially, energy minimization of 1000 steps using the steepest descent algorithm was used, followed by a 200-ps position-constrained MD simulation in order to equilibrate water and ions. After minimization, the systems were gradually heated in the Canonical ensemble from 0 to 300 K in 100 ps using a Langevin thermostat with a coupling coefficient of 1.0/ps and a force constant of 1.0 kcal/mol 3Å on the complex. The system was again equilibrated for 1 ns by removing all restraints. Finally, an MD production run of 20 ns in the Isobaric-Isothermal ensemble was performed. During the simulation, the cut-off distance for the long-range van der Waals (vdW) energy term was set at 10.0 Å. The long-range Coulombic interactions were handled using the particle mesh Ewald (PME) method, and the SHAKE algorithm was applied to all atoms covalently bonded to hydrogen atoms^23^. The simulation was carried out with periodic boundary conditions at constant temperature (300 K) and pressure (1 atm), and an integration time step of 2 fs was used.

### Statistical analysis

Data were presented as mean ± standard deviation (SD). The significance of the difference between groups was evaluated by one-way ANOVA. All *P-*values were two-sided, with *P* < 0.05 considered statistically significant. For SPR, binding curves were fitted into the 1:1 Langmuir binding model, if feasible, and equilibrium dissociation constants (K_D_) were calculated by using BIAevaluation 4.0 software (GE Healthcare).

## Additional Information

**How to cite this article**: Fan, X. *et al*. Bioactivity of 2′-deoxyinosine-incorporated aptamer AS1411. *Sci. Rep.* **6**, 25799; doi: 10.1038/srep25799 (2016).

## Supplementary Material

Supplementary Information

## Figures and Tables

**Table 1:** A list of AS1411 compounds containing 2′-dI with more potent activities.

No	Name	Sequence	K_D_ (nM)	S-Phase (MCF-7)
1.	AS1411	5′-ggt ggt ggt ggt tgt ggt ggt ggt gg	169.0	20.54%
2.	FAN-12dI	5′-ggt ggt ggt gg***dI***tgt ggt ggt ggt gg	/	25.07%
3.	FAN-13dI	5′-ggt ggt ggt ggt***dI***gt ggt ggt ggt gg	/	22.79%
4.	FAN-15dI	5′-ggt ggt ggt ggt tg***dI*** ggt ggt ggt gg	/	25.21%
5.	FAN-24dI	5′-ggt ggt ggt ggt tgt ggt ggt gg***dI***gg	/	21.16%
6.	FAN-1224dI	5′-ggt ggt ggt gg***dI***tgt ggt ggt gg***dI***gg	32.2	33.46%
7.	FAN-1324dI	5′-ggt ggt ggt ggt ***dI***gt ggt ggt gg***dI***gg	30.9	31.12%
8.	FAN-1524dI	5′-ggt ggt ggt ggt tg***dI***ggt ggt gg***dI*** gg	37.0	37.01%
