# Supplementary material for: Bioactivity of 2′-deoxyinosine-incorporated aptamer AS1411
Source: Sci Rep. 2016 May 19;6:25799. doi: 10.1038/srep25799 (PMC4872150; doi:10.1038/srep25799)
Supplement: Supplementary Information [file srep25799-s1.pdf]

Bioactivity of 2'-deoxyinosine-incorporated aptamer AS1411

Xinmeng Fan[1,+], Lidan Sun[2,+], Yun Wu[1], Lihe Zhang[1], Zhenjun Yang[1,*]

[1]State Key Laboratory of Natural and Biomimetic Drugs, School of Pharmaceutical Sciences, Peking University, Beijing 100191, PR China.

[2] Hubei Key Laboratory of Tumor Microenvironment and Immunotherapy, China Three Gorges University & Department of Medical College, Yichang, China.

[+] These authors contributed equally to this paper.

[*] To whom correspondence should be addressed. Tel: 86-10-82802503; Email: yangzj@bjmu.edu.cn

Table S1. A list of AS1411 compounds containing 2’-dI

No	Name	Sequence
1	FAN-3dI	5’-gg*dI*ggt ggt ggt tgt ggt ggt ggt gg
2	FAN-6dI	5’-ggt gg*dI*ggt ggt tgt ggt ggt ggt gg
3	FAN-9dI	5’-ggt ggt gg*dI*ggt tgt ggt ggt ggt gg
4	FAN-14dI	5’-ggt ggt ggt ggt t*dI*t ggt ggt ggt gg
5	FAN-18dI	5’-ggt ggt ggt ggt tgt gg*dI* ggt ggt gg
6	FAN-21dI	5’-ggt ggt ggt ggt tgt ggt gg*dI* ggt gg
7	FAN-alldI	5’-ggt ggt ggt gg*dIdI*g*dI* ggt ggt gg*dI* gg
8	*FAM*- FAN-1524dI	5’-*FAM*-ttt ggt ggt ggt ggt tg*dI* ggt ggt gg*dI* gg

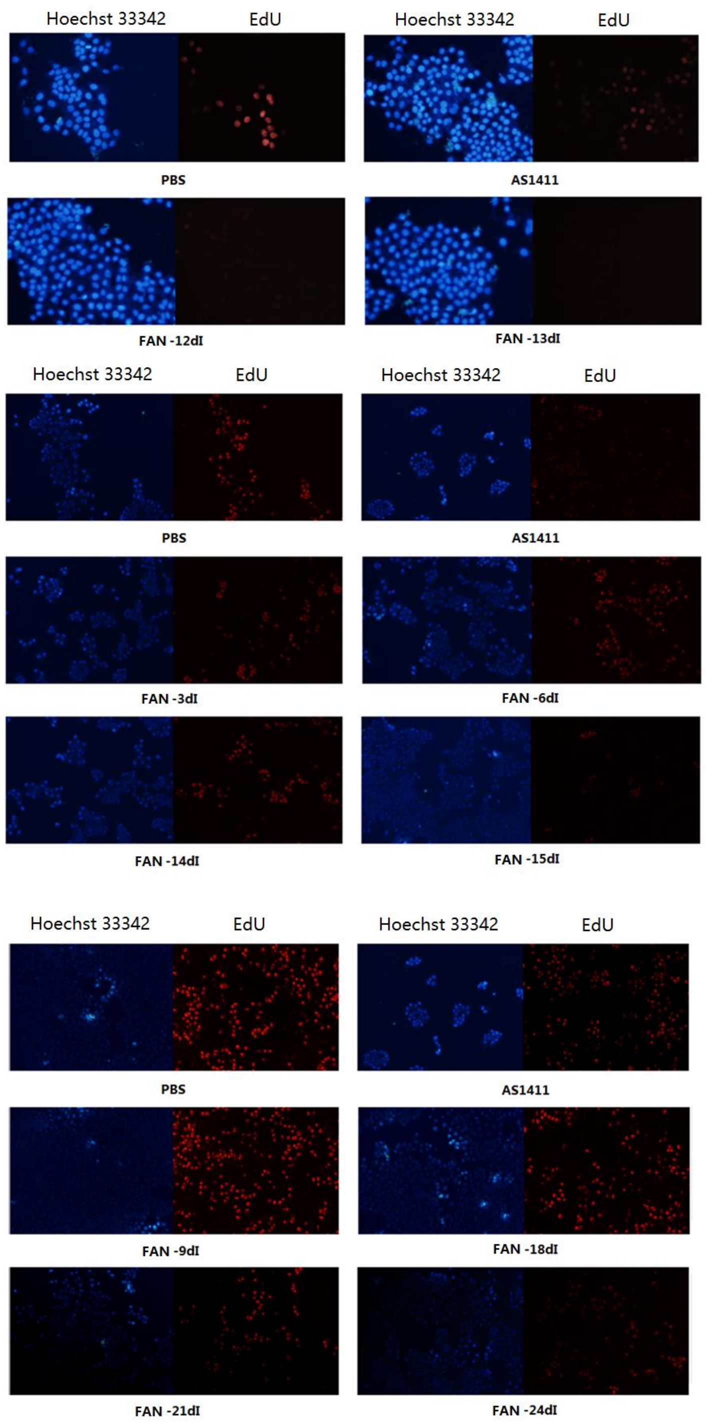

Figure. S1．DNA synthesis in untreated MCF-7 cells (PBS as control) and cells treated with AS1411 (control oligonucleotide) or 2’-dI incorporated AS1411 (active oligonucleotide). Cells were treated a final concentration of 18 μM for 72 h and then expose to 50 μM EdU for 2 h at 37°C.

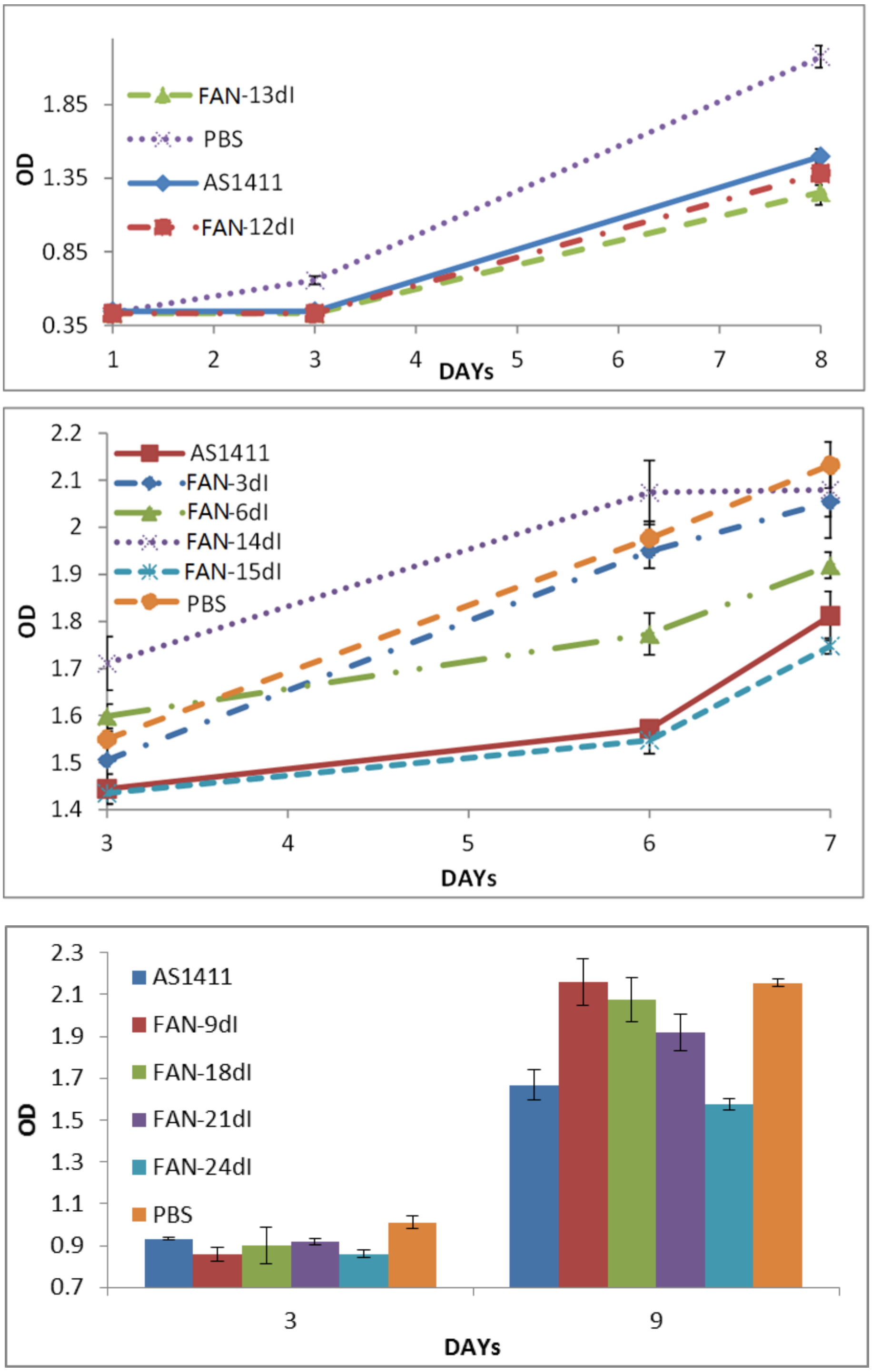

Figure. S2. CCK-8 assays showing the growth of MCF-7 cells treated with 2’-dI incorporated AS1411 or PBS as control. oligonucleotides (or PBS as control) are added directly to the culture medium to give a final concentration of 15 μM (day 1). On days 2-4 further oligonucleotide equivalent to half the initial dose is added. The OD_{450} nm value is proportional tothe number of viable cells in the sample.

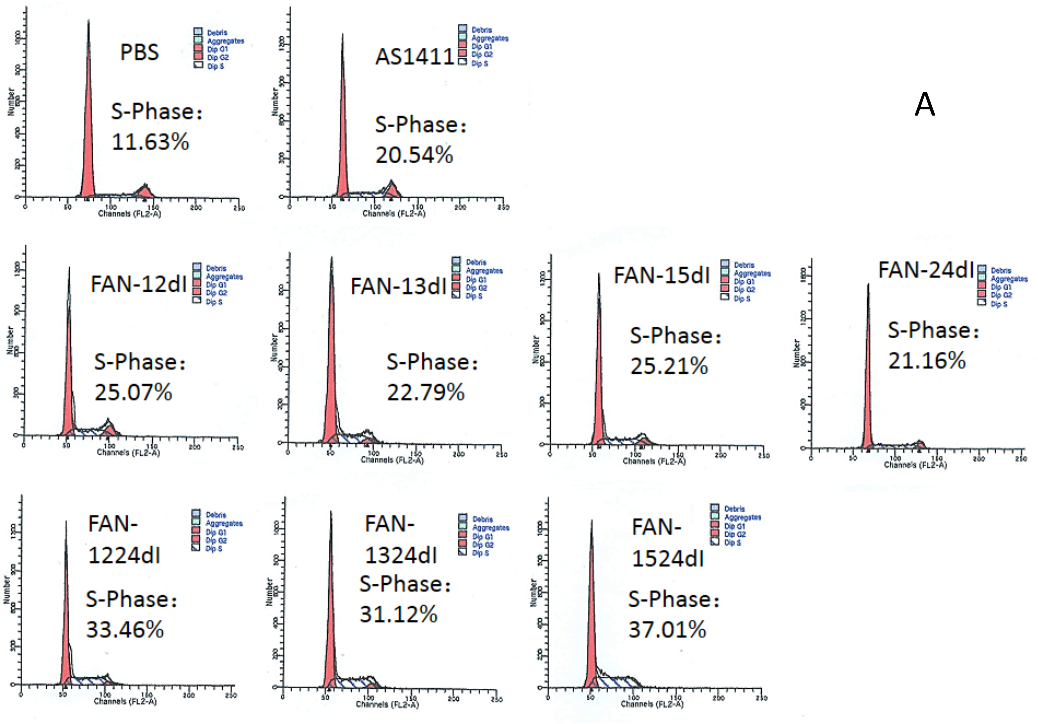

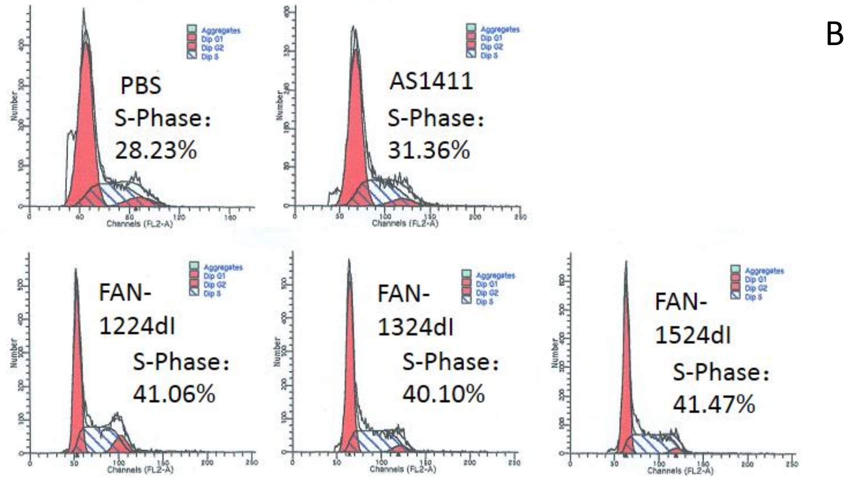

Figure. S3． Flow cytometric analysis of S phase fractions following 72 h of treatment with 2'-dI modified AS1411 or PBS as control. The identity of the cell line is indicatedabove each histogram, cells are treated by direct addition of oligonucleotide to the culture medium to give a final concentration of 10 μM. The percentage of cells in S is determined using the Modfit program. A. MCF-7 cells, B. MDA-MB-231 cells

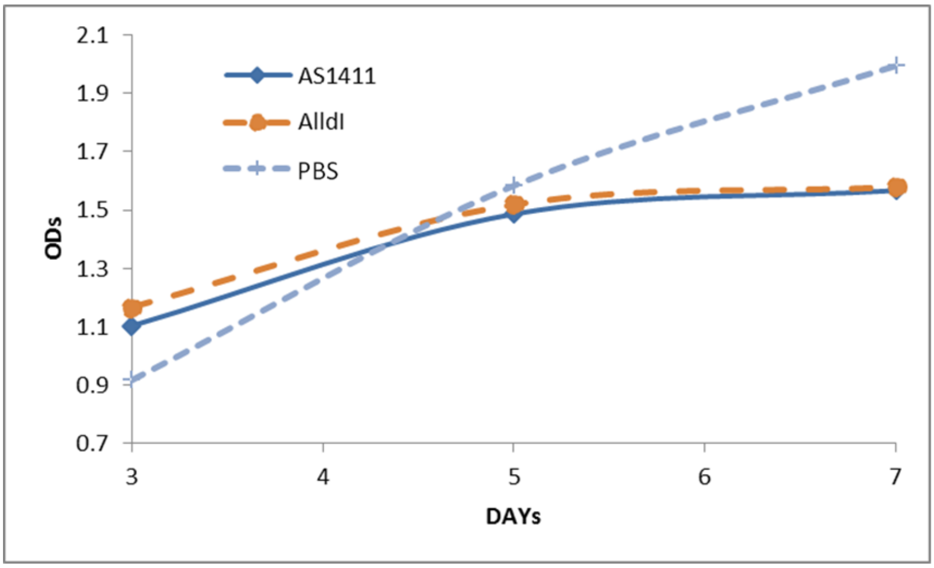

Figure. S4. CCK-8 assays showing the growth of MCF-7 cells treated with AlldI or PBS as control. The numbers of viable cells overtopped than AS1411.

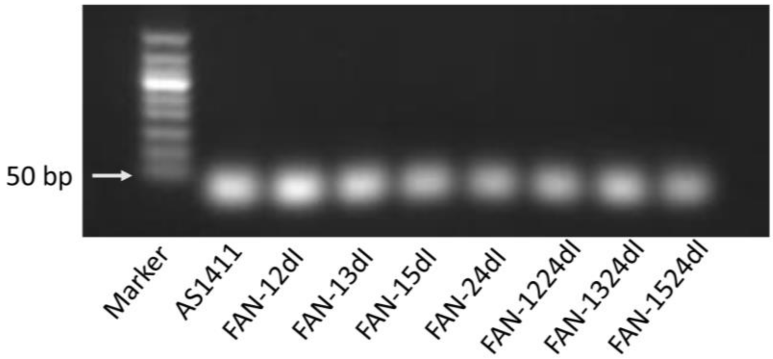

Figure. S5. Aptamers serum degradation assay in serum. Degradation of aptamers expose in DMEM supplemented with 10% fetal bovine serum at 37°C for 3 days, undegraded, intact aptamers were resolved in 20% polyacrylamide gels and SYBR staining.
